# Current and emerging issues in dermatophyte infections

**DOI:** 10.1371/journal.ppat.1012258

**Published:** 2024-06-13

**Authors:** Sarah Dellière, Arnaud Jabet, Alireza Abdolrasouli

**Affiliations:** 1 Service de Parasitologie-Mycologie, Hôpital Saint-Louis, AP-HP, Paris, France; 2 Institut Pasteur, Immunobiology of Aspergillus, Université Paris-Cité, Paris, France; 3 Service de Parasitologie-Mycologie, Hôpital La Pitié-Salpêtrière, AP-HP, Paris, France; 4 Service de Parasitologie-Mycologie, Hôpital Saint-Antoine, AP-HP, Paris, France; 5 Institut Pierre Louis d’Epidémiologie et de Santé Publique, Sorbonne Université, Paris, France; 6 Department of Medical Microbiology, King’s College Hospital, London, United Kingdom; 7 Department of Infectious Diseases, Imperial College London, London, United Kingdom; Vallabhbhai Patel Chest Institute, INDIA

## Introduction

Dermatophyte infections, also known as tinea or ringworm, remain an important public health concern worldwide. Dermatophytes consist of more than 30 species of common and rare contagious filamentous fungi that can invade keratinized tissues and cause superficial infections of the skin, nail, and hair [1]. Infections are acquired from contact with infected humans, animals, soil, or contaminated fomites. Secondary spread from other infected body sites can occur. Infections caused by dermatophytes are considered the most common fungal diseases in humans [2]. It is estimated that more than 20% to 25% of the world population is affected by these superficial fungal skin diseases [3]. The clinical spectrum of dermatophytosis, range from onychomycosis (i.e., nail infection) and tinea pedis (i.e., athlete’s foot) to the inflammatory lesions of tinea corporis and tinea capitis (i.e., skin and scalp ringworm, respectively) [4]. Anthropophilic dermatophyte species usually cause desquamative and pruritic lesions with a low level of inflammation, the most common being *Trichophyton rubrum*, *T*. *interdigitale*, *T*. *tonsurans*, *T*. *violaceum*, *T*. *soudanense*, and *Microsporum audouinii*. In contrast, zoophilic, mostly *T*. *mentagrophytes* and *M*. *canis*, and geophilic species are responsible for more inflammatory lesions when infecting the human host [5]. Dermatophytosis have been commonly managed with terbinafine, this being the first line treatment since the 1990s [6].

The past decade has witnessed the emergence of an increased number of dermatophyte infections with unusual clinical manifestations and/or mode of transmission including **deep** (i.e., invasive) [7], **extensive** (i.e., unusual extent of skin surface or multiple affected sites) [8], **difficult-to-treat** (i.e., not responding to standard course of first line treatment) [9] or **sexually transmitted** dermatophytosis [10]. Herein, we use “complex dermatophytosis” to refer to these infections collectively. The challenges associated with unusual clinical presentations of dermatophytosis include delayed- or mis-diagnosis, leading to unnecessary prescription of antibiotics and/or topical steroids often masking or worsening the preliminary symptoms. Subsequent delayed or ineffective interventions lead to continuous transmission of the disease. Furthermore, unusual **outbreaks of various zoophilic dermatophytes** and their transmission to human populations in recent years is of concern [11,12]. In this rapidly evolving context, we provide an overview of the current clinical challenges in the ever-expanding spectrum of dermatomycology and the dermatophyte pathogens involved. We highlight potential key priorities for future research in the integration and management of complex dermatophyte infections.

### I. Deep dermatophytosis

**Fig 1:**
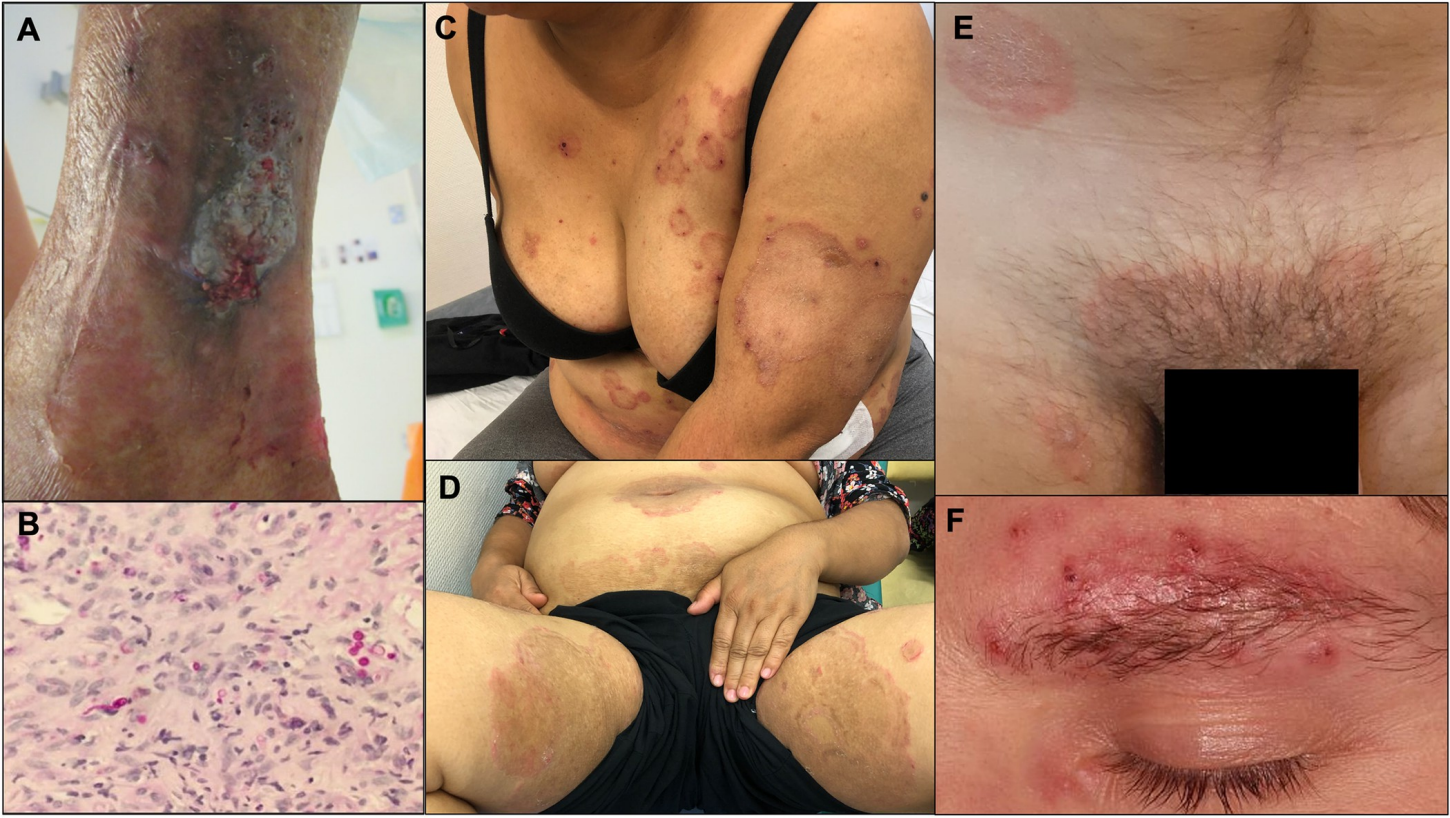
Clinical manifestations of emerging dermatophytosis. (A) Deep dermatophytosis caused by *Trichophyton rubrum* presenting with a nodular lesion on right leg of a renal transplant recipient. (B) Skin biopsy from case A showing granulomatous inflammation with fungal hyphae (dark purple) within the granulomas (PAS stain, ×400); (C) and (D) extensive tinea corporis and tinea crusis due to *T*. *mentagrophytes* complex genotype VIII (*Trichophyton indotineae*); (E) and (F) sexually transmitted tinea corporis due to *Trichophyton mentagrophytes* complex genotype VII.

Overall, the number of invasive fungal diseases are in rise mainly due to wider range and increased number of at-risk immunocompromised populations. Hematopoietic stem cell and solid organ transplantation, and more frequent use of immunosuppressive biologic agents are common risk factors [13]. This also impacts the epidemiology of dermatophytosis, which can become invasive in certain states of immunosuppression and are frequently misdiagnosed by clinicians. Invasive dermatophytosis is defined by dermal invasion presenting as 2 clinical entities: Majocchi’s granuloma and deep dermatophytosis [7]. While Majocchi’s granuloma is limited to perifollicular granulomatous inflammation (presenting as nodules or papules), deep dermatophytosis extends beyond the perifollicular area (with infiltrated, often painful, plaques and nodules) (Fig 1A and 1B). In this latter case, infection can spread to other tissues as seen particularly in CARD9 innate immunodeficiency [14]. Cases of deep dermatophytosis have been especially described in solid organ transplant patients [15,16] but they have also been reported in patients with other immunodeficiencies such as AIDS, immunosuppressive therapy, or hematological disorders [7]. Lesions are usually found on the lower limbs, often in association with superficial dermatophytosis at other body sites [15,16]. Treatment requires prolonged systemic antifungal therapy [15,16].

### II. Extensive dermatophytosis and the emergence of *Trichophyton indotineae*

Extensive dermatophytosis, limited to the stratum corneum, was historically described in immunocompromised patients, especially those receiving corticosteroids [7]. However, it is now increasingly observed in immunocompetent individuals due to a particular dermatophyte clonal species that has emerged recently on the Indian subcontinent and is responsible for outbreaks (Fig 1C and 1D) [8,17]. In 2018, Indian dermatologists warned of the alarming increase in extensive, chronic, and difficult-to-treat anthropophilic dermatophytosis due to the *Trichophyton mentagrophytes* species complex—likely due to widespread misuse of topical antifungals and corticosteroids [17]. Among this species complex, *T*. *interdigitale* is considered anthropophilic and responsible for tinea pedis, while *T*. *mentagrophytes* is considered zoophilic and associated with cases of tinea corporis [18]. The dermatophyte responsible for the Indian outbreak was classified as a particular genotype (i.e., genotype VIII) within the *T*. *mentagrophytes* species complex and its anthropophilic characteristics (human-to-human transmission without direct contact to infected animals) raised a taxonomic debate, which led to the recent naming of a new species, *Trichophyton indotineae* [19]. Although, separating *T*. *indotineae* from *T*. *mentagrophytes* as a new specie is taxonomically inaccurate [18], the new name is clinically relevant and may help awareness. Searching for genotype VIII ITS sequences deposited in GenBank, Jabet and colleagues identified compatible sequences uploaded since 2008, providing evidence that it could have been quietly circulating for more than 10 years [20]. This study also showed that the novel *T*. *indotineae* was present in the Middle East and Southeast Asia, both in humans as well as among animals [20,21]. This has recently been confirmed by 2 studies focusing on animals [22,23]. The recent Indian outbreak has since spread worldwide and cases have been reported on all continents [24,25] with reports from the Americas being much more recent [25–28]. While initial cases were epidemiologically linked to the Indian subcontinent, recent studies have identified instances without any such connection. This raises concerns about the potential local circulation of the pathogen outside the Indian subcontinent including countries not yet on the epidemiological map due to reporting bias [25,27,29]. International travel could therefore be a risk factor for developing an infection. The worrying feature of *T*. *indotineae* infections is that they are difficult to treat, and the standard course of first line treatment is often not sufficient to fully eradicate the infection. In some cases, treatment failure can be explained by the fungus’ *in vitro* resistance to terbinafine and/or azoles (*i.e.*, up to 75% and 25% terbinafine- and itraconazole-resistant isolates, respectively, in certain case series) and linked to various molecular mechanisms (see part III) [8]. However, lack of response to treatment is also probably linked to other clinico-biological factors given the number of relapses despite treatment adapted to the strain’s susceptibility [24,30]. A clinical trial carried out in India studying a treatment regimen of itraconazole to treat tinea corporis caused by itraconazole-susceptible isolates, identified 47.4% (55/126) relapses in patients who completed the study protocol [31]. There is currently no consensus on the most effective treatment approach for infections caused by *T*. *indotineae*, including antifungal agent, dose, duration, and infection control measures required to reduce onward transmission. Early and accurate identification of *T*. *indotineae* as well as antifungal susceptibility testing is essential to initiate prompt and effective antifungal therapy [32,33]. Such investigations should be triggered in case of extensive tinea corporis and/or history of international travel. However, accurate identification of *T*. *indotineae* remains difficult for most routine diagnostic laboratories due to its morphological similarities to other closely related *Trichophyton* species. Antifungal susceptibility testing remains laborious and is discussed below. Several questions need to be addressed urgently to tackle this growing public health issue (Table 1).

**Table 1:** Current gaps and future priorities for research in integration and management of complex dermatophyte infections.

Domain	Current gaps	Research and development priorities
Basic science and epidemiology	• Uncertainties regarding taxonomy and its clinical implications within *T*. *mentagrophytes* species complex • Uncertainties regarding animal or soil reservoirs of *T*. *indotineae* and *T*. *mentagrophytes* ITS genotype VII	• Global epidemiology studies on *T*. *mentagrophytes* species complex using whole-genome sequencing • Taxonomy clarification • Surveys in animal populations • Increase access and training to diagnosis (up to species level) in LMICs
Clinical practice	• Awareness among front-line practitioners regarding the new clinical presentation of complex dermatophytosis	• Promote awareness regarding these new presentations of dermatophytosis through national society channels and public health agencies • Promote best mycological sampling practice
Diagnosis	• Conventional diagnostic methods cannot unequivocally distinguish some dermatophyte species/genotypes • Difficulties in antifungal susceptibility testing	• Sequence-based and rapid molecular methods are needed for accurate identification of closely related species from skin sample • Development and implementation of easy to perform tests for antifungal susceptibility testing • Develop guidelines/algorithms advocating for susceptibility testing
Treatment	• Infection control recommendations to clear *T*. *indotineae* from the environment and avoid spreading to close contacts • Optimize regimen(s) to treat *T*. *indotineae* infections *•* Understanding of origin of frequent relapse in extensive tinea corporis treated with itraconazole	• Evaluate the persistance of *T*. *indotineae* in the environment and study successful methods to eradicate the fungus from the environment and fomites • Clinical trials for the treatment of *T*. *indotineae* infections • Educate patients regarding the risk of relapse and the measures to avoid relapses once they are better understood • Study pharmacokinetic profile of itraconazole while treating *T*. *indotineae* infection
Surveillance	• Clear picture of the ongoing transmission of *T*. *indotineae* cases	• Design and implement national and international surveillance programs to measure the prevalence of various dermatophyte species in humans and animals in line with “One Health” approach • Create a worldwide network to accurately study the yearly incidence of *T*. *indotineae*

LMIC, low and middle income countries.

### III. Resistance to antifungal agents

The emergence and global spread of *T*. *indotineae* has led to a renewed interest in dermatophyte resistance to terbinafine and azoles. The first case of dermatophyte resistance to terbinafine was described in 2003 [34]. Only one of 30 *T*. *rubrum* strains isolated in patients with onychomycosis who had failed 6 months of treatment with oral terbinafine showed increased minimum inhibitory concentration (MIC) to terbinafine and other squalene epoxidase inhibitors. Terbinafine resistance was subsequently associated to point mutations in the *SQLE* gene, encoding squalene epoxidase which is involved in ergosterol biosynthesis [35]. These mutations are concentrated in hotspots leading to substitutions at key positions in the amino acid sequence of the enzyme: 393Leu, 397Phe, 415Phe, 440His. They are found in *T*. *rubrum*, *T*. *interdigitale* as well as *T*. *indotineae*. The most frequently reported substitutions are Phe397Leu and Leu393Phe. They are associated with a significant increase in MICs to terbinafine, unlike other substitutions such as Phe397Ile, Phe415Ile, Phe415Val, and His440Tyr. Outside India, data on terbinafine resistance in dermatophytes are available from Europe, Japan, and the United States of America. The highest rates of resistance were so far reported in the USA, with 4.3% (26/607) of *T*. *interdigitale* isolates and 3.6% (173/4,796) of *T*. *rubrum* isolates found to be resistant to terbinafine [36]. Elevated MICs to azoles are less commonly described and concern especially *T*. *indotineae* isolates. To date, 2 resistance mechanisms have been described: drug efflux via overexpression of ABC-type transporters, and target overexpression via *CYP51* copy number increase [37]. These data call for greater attention to the evaluation of dermatophyte sensitivity to antifungal agents. Antifungal susceptibility testing for *Trichophyton* spp. needs to be performed in routine practice and a standard broth microdilution method has been adapted to the *Trichophyton* genus by the European Committee on Antimicrobial Susceptibility Testing (EUCAST) [32]. Yet, the poor conidiation of certain species (*e.g.*, *T*. *rubrum*) makes it difficult to apply and even for species that conidiate well such as *T*. *mentagrophytes* complex species, this technique remains laborious, time-consuming, and require trained personnel [32]. Terbinafine gradient concentration stripes have been developed and evaluated with unsatisfactory results [38]. Alternatively, medium containing terbinafine can be used to determine if the strain present a wild-type phenotype [39]. However, efforts need to be made to translate *in vitro* MICs into relevant clinical breakpoints for the different species of dermatophytes and to develop cost-effective and less complicated techniques that are easier for nonspecialist laboratories to use to determine sensitivity to antifungal agents.

### IV. Sexually transmitted dermatophytosis

Over the past 20 years, a few publications have provided arguments for considering dermatophytes sexually transmitted, like other skin or hair pathogens (*Sarcoptes scabiei* or *Phtirus pubis*). Indeed, prolonged skin-to-skin contact during sexual contact can promote direct transmission of dermatophytes, as described in combat sports (*i.e.*, tinea gladiatorum). The hypothesis was first put forward in 2002 based on tinea cruris cases in female sex workers [40,41]. Subsequently, some series and case reports have reported cases suspected of being linked to transmission during sexual intercourse [10,42–50]. Arguments in favor of sexual transmission were the location of lesions (genital region, buttocks, face), identification of similar lesions in sexual partners, identification of sexually transmitted infection (STI) risk factors, co-infection with other STIs, unlikely alternative mode of contamination (especially zoonotic). Although various species were identified (*T*. *rubrum*, *T*. *mentagrophytes*, *T*. *indotineae*, *T*. *quinckeanum*, *M*. *canis*), *T*. *mentagrophytes* genotype ITS VII was often incriminated. Several cases were linked to sexual contacts during trips to South-East Asia, notably with sex workers [42,43,45–47]. In 2016/2017, 37 cases were reported in Berlin, in both men and women [43]. Thirteen cases were identified exclusively in men in 2021 to 2022 in Paris [10]. At least 12 patients were identified as men who have sex with men (MSM), 7 were living with HIV and 5 taking HIV pre-exposure prophylaxis. Similar cases reported in Germany in the same population suggest a Europe-wide epidemic [51]. Whole-genome sequencing of the isolates collected from sexual partners would help to confirm this hypothesis. *T*. *mentagrophytes* genotype ITS VII infections were associated with highly inflammatory lesions, including Majocchi’s granuloma-like lesions (Fig 1E and 1F).

Sexually transmitted dermatophytosis is a presumptive diagnosis, since, as with scabies, other modes of infection are possible. Two criteria could be used to identify cases of sexually transmitted dermatophytosis among patients with tinea corporis (including tinea genitalis and tinea cruris) or tinea faciei: (i) STI risk factors in the patient or similar skin lesions in sexual partner(s); and (ii) absence of an obvious alternative cause of dermatophytosis (*e.g.*, contact with infected animals, tinea pedis leading to autoinoculation from feet nails/skin). Dermatophyte lesions in certain body sites (genital region, groin, buttocks, face) are potentially suggestive of sexually transmitted dermatophytosis. Overall, identifying cases of sexually transmitted dermatophytosis enables physicians to propose an STI check-up to the patient and appropriate prevention advice. Conversely, the presence of skin lesions in a patient at risk of contracting an STI should raise the possibility of sexually transmitted dermatophytosis.

### V. The shifting epidemiology of zoonotic dermatophytosis

Zoophilic dermatophytosis varies worldwide, influenced by temporal, sociological, and ecological factors. Factors like trade and animal importation introduce new pathogens to different areas. The true prevalence of infection in animals and humans remains largely unknown due to the lack of molecular-based identification in older studies [52,53]. When transmitted to humans, these dermatophytes primarily affect exposed skin and scalp, causing conditions like tinea corporis and capitis, triggering significant inflammation.

The number of human infections caused by *Trichophyton benhamiae*, usually linked to contact with infected Guinea pigs, has increased significantly in recent years. The infection was first reported in Japan in 2002, subsequently in Germany in 2011 and has since been reported in numerous countries worldwide [54]. In a large German center, *T*. *benhamiae* is the most frequent zoophilic species isolated in human infections, especially in children [55]. The emergence of *T*. *benhamiae* was associated with the high prevalence of this species in Guinea pig in farms and animal shops (between 16.8% and 93.0% according to the study), with more than 90% of infected animals being asymptomatic carriers [12].

Historically, *T*. *quinckeanum* was known to be predominantly associated with mouse favus and was rarely reported in sporadic human infections prior to 2015 [56]. Infections caused by this specie have been increasingly reported in the literature among both humans (causing mainly tinea capitis and corporis) and animals in Germany, Czech Republic [11,57–59], and Iran [60]. Between 2014 and 2021, in Mölbis (Germany), *T*. *quinckeanum* became the third most frequently isolated zoophilic dermatophyte species behind *T*. *benhamiae* and *T*. *mentagrophytes*, ahead of *M*. *canis*, accounting for 19.0% of human zoonotic infections [55]. Recent reports confirmed that in addition to previously recognized reservoirs (*i.e.*, small rodents and camel), other animals including cats, dogs, and horses can be commonly infected and transmit the infection to humans [57,58]. An increase in rodent populations, particularly ground voles, has been proposed as an explanation for the recent increase in incidence of *T*. *quinckeanum* [55].

Recent observations indicate an increase in isolation of zoophilic dermatophytes from human samples during the recent coronavirus pandemic [55]. For example, a significant increase in human infections caused by *T*. *benhamiae* and *T*. *quinckeanum* was noted in Germany when compared to previous years [55]. It could be hypothesized that several factors contributed to these observations including (i) higher adoption of infected asymptomatic animals as companion pets; (ii) reduced access to veterinary clinics to treat infected animals; and (iii) enhanced contact between humans and infected animals (or their contaminated environment and fomites).

It is important to highlight that the majority of these studies have been conducted in Europe. Unfortunately, many countries or centres lack the resources to identify dermatophytes at the species level in routine practice, which likely leads to an underestimation of epidemiological shifts. This underscores the need for improved diagnostic capabilities and more globally representative research in this field.

## Conclusions

Despite the global burden, considerable morbidity, and emerging challenges in the diagnosis and management of infections caused by dermatophytes, they were not included in the recent WHO fungal priority pathogens list [61]. Research on dermatophytes has been hampered by limited funds and lack of awareness in both public and scientific domains leading to delays in both research and development successes compared with those achieved for other more invasive fungal pathogens. We believe urgent attention is needed to recognize the current caveats in our understanding of the epidemiology, evolution, transmission, diagnosis to the specie level, antifungal susceptibility testing, and management of dermatophyte infections affecting human and animal hosts (Table 1). Various key research questions remain to be explored. Collaborative programs between multiple disciplines including dermatology, infectious diseases, microbiology, veterinary medicine, pharmacy, public health, and epidemiology are needed to drive research and facilitate a global response to the changing landscape of dermatophyte infections.
